# The leading causes of death in the US and Mexico’s pediatric population are related to violence: a note on secondary analyses of registered deaths from 2000 to 2022

**DOI:** 10.3389/fpubh.2024.1428691

**Published:** 2024-10-09

**Authors:** Maria F. Castilla-Peon, Pablo L. Rendón, Nadia Gonzalez-Garcia

**Affiliations:** ^1^Hospital Psiquiátrico Infantil Dr. Juan N. Navarro, Servicios de Atención Psiquiátrica, Comisión Nacional de Salud Mental y Adicciones, Mexico City, Mexico; ^2^Institute of Applied Sciences and Technology, National Autonomous University of Mexico, Ciudad de México, Mexico; ^3^Hospital Infantil de Mexico Federico Gomez, Mexico City, Mexico

**Keywords:** adolescent and young adult mortality, violence, mental health, Mexico, suicide, overdose, children mortality, firearm

## Abstract

**Introduction:**

our objective was to analyze the trends in the leading causes of death among the pediatric population aged 1–19 years in Mexico and the United States (US) from 2000 to 2022. Methods. Data for Mexico were sourced from the National Institute of Statistics and Geography (INEGI), while the US data were extracted from the Centers for Disease Control and Prevention’s Wide-ranging Online Data for Epidemiologic Research (CDC-WONDER) databases.

**Results:**

Homicide has been the leading cause of death since 2017 in Mexico and since 2019 in US youths aged 1–19. In Mexico, it reached 6.5 deaths per 100,000 people in 2022. Despite the overall pediatric mortality decline from 2000 to 2022 in both countries, the pediatric homicide rate has increased by 93.3 and 35.8% In Mexico and the US, respectively, and suicide by 86.6 and 36.9%. In both countries, death by firearm-related injuries had risen in a parallel sense. In the US, deaths by drug overdose and poisoning have increased by 314.8%.

**Conclusion:**

Despite advancements in infant healthcare over the past two decades in Mexico, there remains a significant gap in the provision of healthcare services to the adolescent population. Addressing issues related to violence, mental health, and substance abuse through targeted public policies is imperative for both Mexico and the US, especially given their shared border region.

## 1 Introduction

Analysis of the leading causes of death in a population sheds light on priority health issues, raising a warning flag on structural problems within the systems tasked with ensuring the well-being of people. The World Health Organization (WHO) has expressed concern regarding the high proportion of deaths attributable to firearms and interpersonal violence among the young population in the Americas (1). For example, the United States (US), a high-income country sharing a border with Mexico, has reported a notable increase in mortality due to homicides, suicides, and substance overdoses in its pediatric population (2, 3). There is insufficient assessment of these issues in Mexico.

A comparative analysis of the leading causes of death in the pediatric population through time in neighboring countries reflects the evolving nature of social determinants of health and the regional interdependence of some of these factors. The US Surgeon General Advisory has recently declared firearm violence a public health crisis in this country highlighting the three-decade rise in death rate by this means (4). A similar analysis of the causes of death in the pediatric population has not been thoroughly explored in Mexico in recent years. These two countries have many social, cultural, and economic differences. However, by sharing a border, they have common problems, such as the availability of firearms and drugs and the violence that goes with it. The CDC has proposed that the approach to preventing violence should be a public health strategy, with the first step being spatial and temporal monitoring of the problem (5).

This study’s objective was to describe the trends in the 10 leading causes of death in the Mexican pediatric population aged 1–19 years over the past two decades, with a particular interest in violence and mental health-related deaths. Besides, it aims to compare the mortality trends in Mexico with the trends in the US and to describe mortality by substance overdose, which has emerged as an important cause of death in US youths.

## 2 Methods

### 2.1 Study design and setting

This descriptive study analyzes mortality data in the pediatric Mexican and US populations from 2000 to 2022.

### 2.2 Study population

The child group comprised youths aged 1 to 9 years old, and the adolescent group from 10 to 19 years old.

### 2.3 Variables

We selected the causes of death following the United Nations 2030 Health Sustainable Development Goals indicators on communicable and non-communicable diseases, injuries, and COVID-19 (6). We obtained a list of 24 causes of death (Supplementary Table S1) and selected the top ten causes of death in Mexico. We grouped causes of death according to the International Classification of Diseases 10th revision (ICD-10) (7) codes outlined in Supplementary Table S1. We used de same grouping of codes as Cunningham et al. to get comparable results (2). Included groups were homicide, transport accidents, malignant neoplasms, suicide, congenital anomalies, cardiovascular diseases, neurological disorders, lower respiratory infections, cardiovascular diseases, COVID-19, and drug overdose. We included an additional overlapping group of “firearm injuries related deaths,” which includes those by homicide, suicide, accidental, and unknown intention. Of note, the category “Drug overdose and poisoning” includes codes for any drug and any intentionality. The mortality data correspond to the primary cause of death as recorded on death certificates.

### 2.4 Data sources

We extracted data reported by the National Institute of Statistics and Geography (INEGI) (8, 9) for the Mexican population and by the Centers for Disease Control and Prevention’s Wide-ranging Online Data for Epidemiologic Research (CDC-WONDER) databases for the US population (10).

### 2.5 Statistical methods

Mortality rates are reported as deaths per 100,000 people. We conducted a *post hoc* exploratory analysis by linking municipal death rates to the municipal poverty rates and male adolescents school attendance rates, as reported by the National Council of Development Politics Evaluation (CONEVAL) in 2020 (11), along with the number of organized crime cartels reported in each state (12). This analysis included 1,852 out of 2,469 municipalities with a population of over 1,600 inhabitants under 20 years old. Municipalities with smaller populations were excluded due to inflated death rates observed in nine of these locations, which had reported casualties. Associations with poverty and male adolescent school attendance rate deciles were expressed as adjusted Incidence Rate Ratios (IRR), with 95% Confidence Intervals, estimated using a Negative Binomial Regression model.

Statistical analyses and graphical representation were done with R version 4.1.2 (R Project for Statistical Computing, Vienna, Austria) and Stata/MP, version 14.0 (StataCorp, College Station, TX, United States).

## 3 Results

Between 2000 and 2022, Mexico recorded 511,396 deaths among individuals aged 1–19 years. Over this period, there was a 9.4% reduction in the all-cause mortality rate, declining from 53.1 to 48.1 deaths per 100,000 people. Since 2017, homicides have been the leading cause of death among Mexican children and adolescents (6.5 deaths per 100,000 in 2022), constituting 13.4% of all deaths in 2022. That year, motor vehicle accidents were the second leading cause (11.5% of all deaths), followed by other non-communicable diseases such as cancer (10.4%), neurological disorders (7.9%), congenital anomalies (7.7%), non-transport accidents (6.9%) and suicide (5.6%). Lower respiratory infections (4.3%), cardiovascular diseases (3.9%), and COVID-19 (2%) were the sixth to ninth leading causes of death.

**Figure 1:**
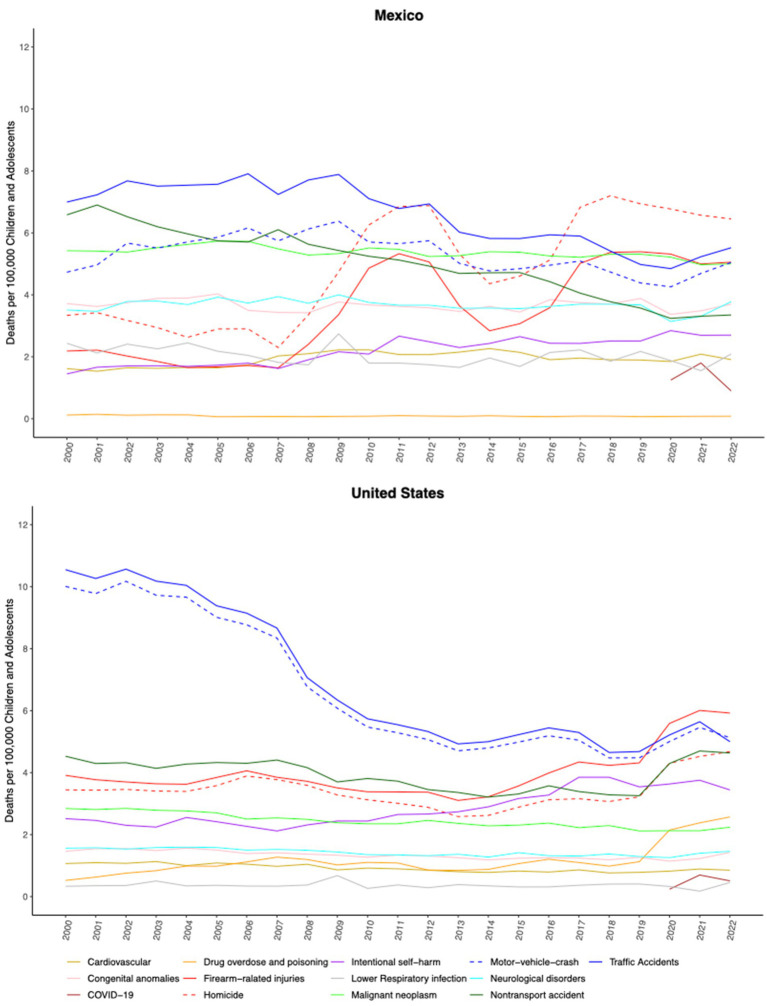
The leading causes of death in children and adolescents aged 1–19 years in Mexico and the United States (US) from 2000 to 2022. Firearm-related injuries include homicides, self-harm, and unintentional injuries.

**Figure 2:**
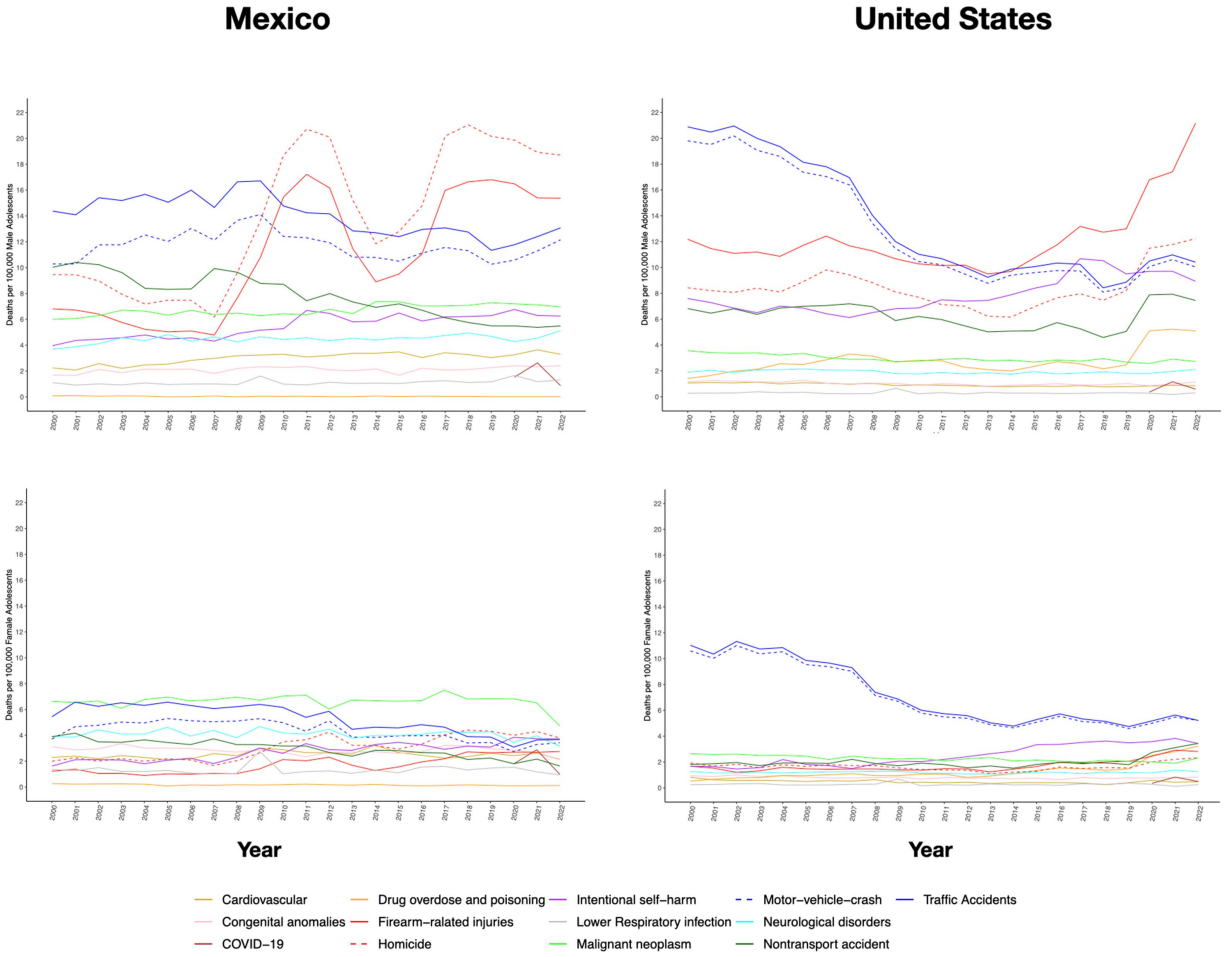
The leading causes of death in adolescents aged 11–19 years in Mexico and the United States (US) by sex from 2000 to 2022. Firearm-related injuries include homicides, self-harm, and unintentional injuries.

While death due to diarrheal diseases, lower respiratory infections, and non-transport injuries have declined in the Mexican population aged 1–19 years, the rates for 3 out of the 13 analyzed diagnostic categories—homicide, firearm-related injuries (which are predominantly subsumed under the former), and suicide—increased significantly from 2000 to 2022. Regarding homicides, which ranked as the leading cause of death in this age group, the rate doubled from 3.3 to 6.5 homicides per 100,000 individuals in Mexico, similar to what is observed in the US, which increased its rate from 3.8 to 4.7 homicides per 100,000. Similarly, deaths by firearms rose markedly in both countries, by 120.7% in Mexico (from 2.2 to 4.9 per 100,000) and by 51.4% in the US (from 3.9 to 5.9 per 100,000) (Table 1). The male adolescent population is the most affected by violent deaths on both sides of the border, with rates of 18.7 and 12.2 homicides per 100,000 male adolescents in Mexico and the US in 2022, respectively. Notably, female Mexican adolescents also exhibited elevated homicide rates, reaching 4.3 and 2.7 per 100,000 people, respectively (Figures 1, 2 and Supplementary Table S6).

**Table 1:** The death rate per 100,000 people for children and adolescents aged 1–19 years in Mexico and the United States, 2000 versus 2022, for the nine leading causes of death in Mexico, plus deaths due to COVID-19, drug overdose, and firearms.

	Mexico			United States		
	2000	2022	Change	2000	2022	Change
All causes	53.1	48.1	−9.4%	33.9	29.9	−11.7%
Firearm-related injury*	2.2	4.9	120.7%	3.9	5.9	51.4%
Homicide	3.3	6.5	93.3%	3.8	4.7	35.8%
Self-harm	1.4	2.7	86.6%	2.5	3.44	36.9%
Neurological disorders	3.5	3.8	7.8%	1.6	1.5	−6.4%
Motor vehicle crash	4.7	4.8	2.4%	10.0	5.13	−48.8%
Congenital anomalies	3.7	3.7	−0.4%	1.5	1.2	−2.4%
Malignant neoplasm	5.4	5.0	−7.7%	2.8	2.2	−21.3%
Cardiovascular diseases	1.6	1.9	18.8%	1.1	0.85	−19.8%
Lower respiratory infections	2.4	2.0	−18.0%	0.4	0.5	26.3%
Non-transport accidents	6.6	3.2	−51.2%	4.5	4.6	2.4%
Drug overdose	0.1	0.0	−59.2%	0.5	2.6	314.8%
COVID-19	–	0.9	–	–	0.5	–

^*^Includes homicides, self-harm, and non-intentional injuries.

Red: Higher death rates/Death rate increase. Blue: Death rate decrease.

Regarding suicide in the population aged 1–19 years, the rate has surged by 86% in Mexico (from 1.4 to 2.7 deaths per 100,000 people) and by 49.3% in the US (from 2.5 to 3.8 per 100,000 people). Table 1 outlines mortality from other leading causes of death in Mexico and the US and their relative variation from 2000 to 2021. Extensive data can be found in the Supplementary file.

In the US population aged 1–19 years old, deaths from drug overdose have increased sharply by 356% over the last two decades, rising from 0.5 to 2.4 per 100,000 people. This surge has been particularly pronounced among female adolescents, soaring from 0.52 to 2.81 (a 436% increase). As of 2022, Mexico had not registered a similar rise in deaths due to overdose.

In Mexico, the number of active organized crime cartels in each state varies from 1 to 15 (12). Homicide rates were positively associated with the number of active drug cartels documented in the state with a crude IRR of 2.4 (95% CI:1.7, 3.3) in states with 3 or more active cartels compared to those with one or two. According to CONEVAL data (11), the median municipal poverty rate in children and adolescents is 70% (IQR: 54–85.5%), and the median school enrollment rate in 15 to 17-year-old male adolescents is 66.9% (IQR: 57.4–75.0%). Poverty and school enrollment rates in male adolescents were both inversely related to homicide rates in municipalities. The negative binomial regression model showed a 20% decrease in homicide rate for each higher decile in adolescent male school enrollment (adjusted IRR = 0.8, 95% CI: 0.74–0.87) and a 14% lower mortality rate associated with each decile increase in poverty rate adjusted IRR = 0.76 (95% CI, 0.70, 0.83). Supplementary Table S13 reports the full model.

## 4 Discussion

This study aimed to quantify the size of health problems in the pediatric population in terms of mortality and shows the absolute impact of selected causes of death and their relative impact compared to previous years and to other health problems. According to this data, the health issues that have the most significant impact on pediatric mortality are related to violence and mental health disorders both in the US and in Mexico.

Despite the decline in all-cause mortality and mortality from communicable diseases in the pediatric population, violent deaths—including those resulting from homicide, firearm-related injuries, and suicide—have alarmingly increased over the last decade in both Mexico and the US. These emerging problems predominantly affect adolescents in both countries. Additionally, the US has experienced a sharp rise in deaths due to drug overdose and poisoning, a phenomenon not registered in Mexico as of 2022. These emerging problems predominantly affect adolescents in both countries.

This study’s data highlights three issues that deserve attention in research and public policy development: (1) the international public health implications of violence, (2) the burden of these problems specifically on the adolescent population, and (3) the need for a cooperative strategy of the two neighboring countries for the maintenance of their youth wellbeing.

Violence and mental health are highly complex phenomena. Social scientists have done extensive research to describe them and address their causes. Studies have shown that socially vulnerable communities disproportionately bear the burden of assault-related firearm deaths among youths. The factors that contribute to this violence include poverty, underfunded public housing, inadequate public services, struggling schools, limited job opportunities, and a pervasive sense of hopelessness (13–16). The interrelatedness of drug overdose and firearm-related injuries has been suggested in ecological studies (17).

It is known that the primary risk factor for the occurrence of firearm-related injuries in children is the availability of firearms at home, and there is an inverse relationship between the stringency of firearm laws and pediatric firearm mortality in the US (18). Of note, despite Mexico’s stringent firearms laws, illegal arms trafficking remains pervasive, and these arms come primarily from the US (19).

**Figure 3:**
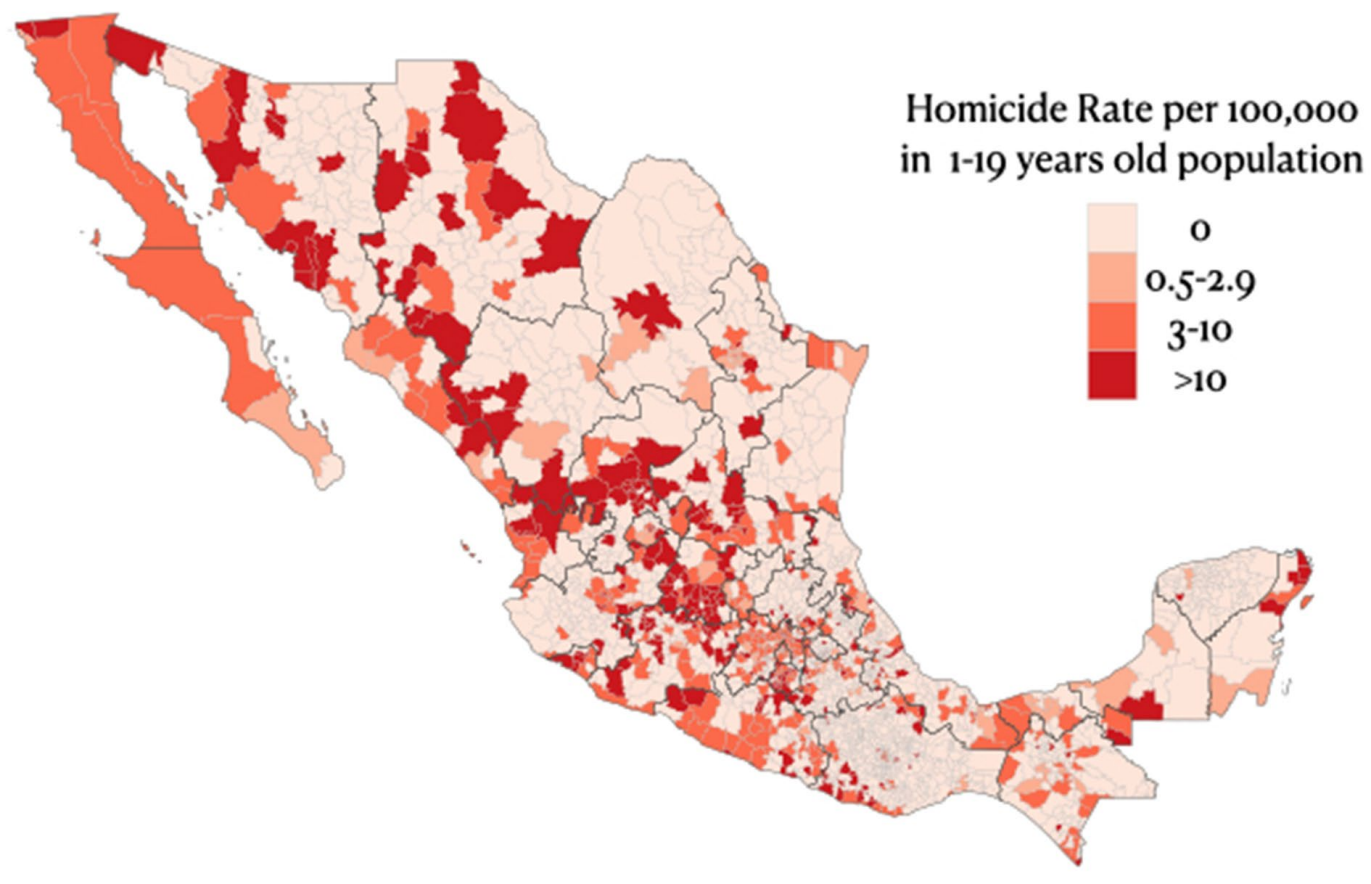
Mortality per 100,000 people aged 1-19 in Mexico in 2022, by municipality.

Violence in Mexico has been primarily attributed to criminal activities related to the illicit drug trade and organized crime. The peak in homicides in 2007–2008 coincides with the Mexican government’s launch of the “War against Narco” strategy (19). Drug-related criminality in Mexico cannot be fully understood without considering two factors originating in the US: the high rate of drug abuse among the US population, and the high flow of US-produced firearms across the Mexican border (20, 21). The inverse relationship between poverty and violent deaths, as well as between adolescent male school enrolment and homicide rates in Mexico is unlikely to be causal. Instead, it might reflect the decision of young males to engage in criminal activities as a means of improving their economic and social status in the absence of better opportunities. (Figure 3).

Suicide is another escalating cause of mortality in the pediatric population. In the US, the surge in suicidal behavior has been linked to a mental health crisis characterized by a rising prevalence of anxiety and depressive disorders (22). In Mexico, a 3.4-fold increase in the rate of lifetime suicide attempts among adolescents from 2006 to 2018 has been reported (23), and a further rise since then can be observed in our data. More research is necessary to understand the social and structural determinants of mental health in Mexico and the US to implement prevention and promotion strategies tailored to children and adolescents.

The death rate due to drug overdose and poisoning in the US adolescent population has alarmingly increased over the last few years. This category includes codes for intentional, accidental, and undetermined intent poisoning by “narcotics and hallucinogens,” as well as codes for other drugs. Of note, “accidental drug overdose or poisoning by narcotics and hallucinogens” was the most frequent subcategory and the one responsible for the rise in deaths by overdose/poisoning. Unfortunately, these codes do not differentiate between prescription vs. illicit narcotics. However, it has been suggested that the availability of prescription opioid analgesics might be a risk factor for illegal opioid consumption (24). The US is one of the countries with the highest opioid analgesics consumption, with about 25 times the worldwide average consumption and about 500 times the amount in Mexico (25). More stringent criteria for opioid analgesic prescription in the US might be a necessary policy to reduce the opioid abuse disorder that leads to illegal opioid demand in this country.

The disproportionately high mortality rate among male adolescents due to intentional and unintentional injuries underscores the urgency to explore further the relationship between gender, violence, and risk behaviors. Moreover, it emphasizes the inadequate coverage of adolescents’ healthcare needs, reflected, for example, in the absence of formal graduate programs for medical specialization in adolescent medicine in Mexico and the lack of efforts to overcome barriers to healthcare access specific to this population.

This study’s limitations include its reliance on available databases. A major issue in Mexico is the relatively large number of missing people: in 2022, there were 309 officially reported cases of missing youths aged 1–19 years because of involuntary or unknown reasons (0.76 per 100,000 people). A portion of these missing people may have died (26).

Conclusions. Homicide, suicide, drug abuse, and firearm usage are the most significant issues in pediatric health in terms of mortality. The violence and mental health problems behind this phenomenon are multifaceted issues that are deeply entrenched in geopolitical, economic, and sociological contexts. The data presented in this report underscore the need for research on the social determinants of these health problems and the development of public health policies aimed at mitigating the exposure of children to violence, the enhancement of adolescent care, limiting firearm availability, reducing unnecessary opioid analgesic prescriptions, and bolstering mental health services. These measures would align with the recommendations outlined in the United Nations Sustainable Development Goals for 2030. While efforts to address these challenges through national security and judicial avenues are necessary, they are insufficient. It is essential to engage other sectors, including the health and education systems, and to foster multinational cooperation to curb childhood and adolescent mortality rates while enhancing their overall well-being. Health professionals should counsel families and youths on violence and mental health issues and turn into advocates for bi-national policies aimed at reducing violence and caring for the mental health of youths to lower crime, suicide, and illicit drug consumption.

## Data Availability

The original contributions presented in the study are included in the article/Supplementary material, further inquiries can be directed to the corresponding author.
