# Supplementary material for: The leading causes of death in the US and Mexico’s pediatric population are related to violence: a note on secondary analyses of registered deaths from 2000 to 2022
Source: Front Public Health. 2024 Oct 9;12:1428691. doi: 10.3389/fpubh.2024.1428691 (PMC11496252; doi:10.3389/fpubh.2024.1428691)
Supplement: Supplementary file 1 [file Data_Sheet_1.DOCX]

**The leading causes of death in the US and Mexico´s pediatric population are related to violence: a note on secondary analyses of registered deaths from 2000 to 2022**

Maria F. Castilla-Peon, MSc^1^. Pablo L. Rendón, PhD^2^ , Nadia Gonzalez-Garcia, PhD^3*^.

**Supplementary tables**

**Table of contents**

1. **International Classification of Diseases, 10^th^ version codes for the Leading Causes of Death among Children and Adolescents in Mexico, Brazil, and the United States used to calculate death rates by cause……………...1**
2. **Population of children adolescents 1-19 years old, and deaths by cause from 2000 to 2022 in Mexico………….2**
3. **Population of male children 1-9 years old, and deaths by cause from 2000 to 2022 in Mexico…………………. 3**
4. **Population of female children 1-9 years old, and deaths by cause from 2000 to 2022 in Mexico………………...4**
5. **Population of male adolescents 10-19 years old, and deaths by cause from 2000 to 2022 in Mexico…………….5**
6. **Population of female adolescents 10-19 years old, and deaths by cause from 2000 to 2022 in Mexico…………. 6**
7. **Population of children adolescents 1-19 years old, and deaths by cause from 2000 to 2022 in the US…………..7**
8. **Population of male children 1-9 years old, and deaths by cause from 2000 to 2022 in the US……………….…..8**
9. **Population of female children 1-9 years old, and deaths by cause from 2000 to 2022 in the US ………………...9**
10. **Population of male adolescents 10-19 years old, and deaths by cause from 2000 to 2022 in the US………….…10**
11. **Population of female adolescents 10-19 years old, and deaths by cause from 2000 to 2022 in the US…………..11**
12. **Supplementary Table 12. Negative Binomial Regression Model to assess the association of municipal poverty rate in children and adolescents, and 15-17 male school enrolment rate with homicide rate in the pediatric population in Mexico in 2022. …………………………………………………………………………………………………….12**

| ^Cause of Death^ | ^ICD-10 Codes^ |
| --- | --- |
| ^1.Lower Respiratory infection^ | ^J09-J18; J20-22^ |
| ^2.Tuberculosis^ | ^A15-19, J65, B90^ |
| ^3.Acute gastroenteritis^ | ^A00-A09^ |
| ^4.Vaccine preventable Disease^ | ^A33, A35,A36-A37, A80, B01, B05, B03, B06,B26, B91^ |
| ^5.Meningitis/encephalitis^ | ^A39, A81, A83-A89, G00-G09^ |
| ^6. Human Immunodefficiency Virus infection^ | ^B20-B24, C46, D84, R75^ |
| ^7.Acute bacterial sepsis and^  ^Severe infections^ | ^A20-A28, A32, A38, A40-A49, A68, A70, A74-A79, B95-B96, H10, H60, I30, I32-I33, I39- I41, K02, K04-K05, K61, K65, K67, K81, L00-L04, L08, M00-M01, M60, M86, N10, N30, N34, N41, N49, N61, P36-P39, U80-U89^ |
| ^8.Other infectious and parasitic disease^ | ^A30, A31, A50, A51-A53, A54-A64, A65-A67, A69, A71, A82, A90-A99, B00 B02 B04 B07- B09 B15-B17 B19 B25 B27-B49 B55-B58 B59, B60/B64 B65-B83, B85-B89, B92, B94, B97- B99, N70-N74, P35, Y95^ |
| ^9. and Perinatal conditions^ | ^O00-O99, A34, F53; P00-P22, P24-P29, P50-P96^ |
| ^10.Malnutrition^ | ^E40-E46^ |
| ^11.Congenital Anomalies^ | ^Q00-Q99^ |
| ^12.Neurological Disorder^ | ^G00-G99^ |
| ^13.Malignant neoplasm^ | ^C00-C97^ |
| ^14.Cardiovascular^ | ^G45-G46, G81-G83, I00-I28, I31, I34-I38, I42-I84, I86-I99, R00-R01, R03, R55^ |
| ^15.Diabetes, Endocrine and^  ^Immune disorders^ | ^E10-E14, D55-D63, D64, D65-D83, D86-D89, E03-E07, E15-E16, E20-E35, E65-E90, R70- R74, R76, R77, R81^ |
| ^16.Chronic respiratory diseases^ | ^J30-J31, J33-J35, J37-J64, J66-J84, J90-J99, R04-R06, R84, R91^ |
| ^17.Homicide^ | ^*U01-*U02 (terrorism), X85-Y09, Y87.1 (homicidal)^ |
| ^18.Firearm Injuries Related^ | ^W32- W34 (accidental); X72-74 (self-harm); X93-X95 (homicide); Y22-Y24 (undetermined intention)^ |
| ^19.Motor Vehicle Crash^ | ^V02-V04, V09.0, V09.2 (pedestrian), V12-V14, V19.0-V19.2, V19.4-V19.6 (pedal cyclist), V20-V79, V80.3-V80.5 V81.0-V81.1, V82.0-V82.1, V83-V86, V87.0-V87.8, V88.0-V88.8, V89.0, V89.2 (occupant of motor vehicle)^ |
| ^20.Transport Accidents^ | ^V01-V99,Y85^ |
| ^21.Intentional Self-harm^ | ^*U03 (terrorism), X60-X84,Y87.0^ |
| ^22.Non-transport accident^ | ^W00-X59, Y86^ |
| ^23.Drug Overdose and poisoning^ | *^X40-44 (^*^accidental^*^) ; X60-64(^*^self-harm)^ *^; X85^* ^(homicidal)^*^; Y10-Y14^* ^(undetermined intention)^ |
| ^24.COVID-19^ | ^*U07.1 and U07^ |

**Supplementary Table 1.** **International Classification of Diseases, 10^th^ version codes for the Leading Causes of Death among Children and Adolescents in Mexico and the United States used to calculate death rates by cause.**

**Supplementary Table 2. Population of children and adolescents 1-19 years old and deaths by cause from 2000 to 2022 in Mexico**

|  | Population | Total Deaths | Homicides | Firearm-related injuries | Transport accidents | Motor vehicle crash | Malignant neoplasm | Cardiovascular | Congenital anomalies | Non- transport accidents | Neurological disorder | Intentional self-harm | Lower respiratory infection | Acute gastroenteritis | Malnutrition | COVID-19 |
| --- | --- | --- | --- | --- | --- | --- | --- | --- | --- | --- | --- | --- | --- | --- | --- | --- |
| 2000 | 40517677 | 21527 | 1352 | 886 | 2834 | 1917 | 2198 | 656 | 1506 | 2667 | 1423 | 587 | 987 | 903 | 611 | .. |
| 2001 | 40392581 | 21226 | 1383 | 895 | 2920 | 2006 | 2186 | 620 | 1464 | 2787 | 1400 | 672 | 858 | 812 | 485 | .. |
| 2002 | 40267485 | 21532 | 1278 | 815 | 3092 | 2284 | 2166 | 663 | 1511 | 2626 | 1525 | 687 | 970 | 833 | 561 | .. |
| 2003 | 40142388 | 20989 | 1181 | 742 | 3014 | 2211 | 2217 | 654 | 1560 | 2488 | 1525 | 687 | 906 | 812 | 541 | .. |
| 2004 | 40017292 | 20617 | 1052 | 661 | 3017 | 2285 | 2252 | 664 | 1559 | 2388 | 1475 | 677 | 980 | 755 | 476 | .. |
| 2005 | 39892196 | 20787 | 1156 | 669 | 3020 | 2337 | 2288 | 656 | 1608 | 2291 | 1566 | 690 | 869 | 789 | 435 | .. |
| 2006 | 40225508 | 20437 | 1167 | 692 | 3181 | 2478 | 2304 | 698 | 1408 | 2295 | 1502 | 722 | 823 | 610 | 376 | .. |
| 2007 | 40558821 | 20628 | 932 | 667 | 2937 | 2329 | 2224 | 822 | 1392 | 2475 | 1599 | 658 | 738 | 623 | 416 | .. |
| 2008 | 40892133 | 20823 | 1372 | 983 | 3152 | 2505 | 2161 | 859 | 1399 | 2303 | 1525 | 779 | 712 | 601 | 414 | .. |
| 2009 | 41225446 | 22389 | 1955 | 1385 | 3251 | 2629 | 2198 | 917 | 1553 | 2239 | 1649 | 892 | 1130 | 457 | 426 | .. |
| 2010 | 41558758 | 22044 | 2599 | 2020 | 2953 | 2371 | 2291 | 924 | 1527 | 2180 | 1561 | 868 | 747 | 391 | 395 | .. |
| 2011 | 41477782 | 21963 | 2841 | 2210 | 2815 | 2345 | 2268 | 860 | 1505 | 2125 | 1520 | 1106 | 746 | 432 | 383 | .. |
| 2012 | 41396806 | 21402 | 2847 | 2095 | 2871 | 2379 | 2168 | 857 | 1483 | 2040 | 1518 | 1029 | 722 | 402 | 307 | .. |
| 2013 | 41315830 | 19877 | 2198 | 1506 | 2488 | 2073 | 2175 | 889 | 1432 | 1938 | 1474 | 950 | 685 | 417 | 366 | .. |
| 2014 | 41234854 | 19323 | 1798 | 1171 | 2400 | 1968 | 2223 | 934 | 1496 | 1943 | 1474 | 1003 | 809 | 389 | 364 | .. |
| 2015 | 41153878 | 19086 | 1895 | 1263 | 2394 | 1993 | 2212 | 882 | 1418 | 1942 | 1463 | 1091 | 695 | 384 | 322 | .. |
| 2016 | 41072901 | 19,376 | 2106 | 1474 | 2439 | 2036 | 2158 | 783 | 1579 | 1817 | 1490 | 1001 | 879 | 426 | 300 | .. |
| 2017 | 40991925 | 20164 | 2799 | 2057 | 2417 | 2087 | 2137 | 803 | 1536 | 1662 | 1516 | 997 | 911 | 302 | 322 | .. |
| 2018 | 40910949 | 19567 | 2945 | 2197 | 2215 | 1938 | 2172 | 779 | 1516 | 1546 | 1513 | 1027 | 759 | 290 | 301 | .. |
| 2019 | 40829973 | 19470 | 2833 | 2201 | 2033 | 1790 | 2169 | 774 | 1586 | 1459 | 1501 | 1025 | 887 | 359 | 282 | .. |
| 2020 | 40748997 | 18767 | 2759 | 2166 | 1975 | 1735 | 2126 | 753 | 1373 | 1320 | 1280 | 1159 | 765 | 301 | 183 | 509 |
| 2021 | 40668021 | 19206 | 2672 | 2036 | 2127 | 1911 | 2023 | 848 | 1417 | 1346 | 1345 | 1096 | 631 | 312 | 220 | 732 |
| 2022 | 40587045 | 19537 | 2618 | 2054 | 2240 | 2048 | 2032 | 775 | 1503 | 1358 | 1536 | 1097 | 847 | 291 | 219 | 379 |

**Supplementary Table 3. Population of 1-9 years old male children and deaths by cause from 2000 to 2022 in Mexico**

| \|  \| Population \| Total Deaths \| Homicides \| Firearm-related injuries \| Transport accidents \| Motor vehicle crash \| Malignant neoplasm \| Cardiovascular \| Congenital anomalies \| Non- transport accidents \| Neurological disorder \| Intentional self-harm \| Lower respiratory infection \| Acute gastroenteritis \| Malnutrition \| COVID-19 \| Drug Overdose \| \| --- \| --- \| --- \| --- \| --- \| --- \| --- \| --- \| --- \| --- \| --- \| --- \| --- \| --- \| --- \| --- \| --- \| --- \| \| 2000 \| 11077853 \| 5751 \| 122 \| 46 \| 591 \| 337 \| 532 \| 112 \| 563 \| 830 \| 427 \| 8 \| 414 \| 395 \| 252 \| .. \| 8 \| \| 2001 \| 10964841 \| 5588 \| 121 \| 43 \| 580 \| 345 \| 559 \| 106 \| 575 \| 859 \| 408 \| 2 \| 354 \| 372 \| 198 \| .. \| 10 \| \| 2002 \| 10851980 \| 5474 \| 102 \| 38 \| 622 \| 417 \| 529 \| 109 \| 556 \| 852 \| 425 \| 7 \| 382 \| 369 \| 225 \| .. \| 5 \| \| 2003 \| 10738986 \| 5474 \| 92 \| 37 \| 585 \| 349 \| 535 \| 116 \| 592 \| 802 \| 408 \| 0 \| 399 \| 353 \| 200 \| .. \| 8 \| \| 2004 \| 10625915 \| 5373 \| 65 \| 26 \| 559 \| 357 \| 541 \| 113 \| 581 \| 814 \| 404 \| 0 \| 404 \| 330 \| 206 \| .. \| 6 \| \| 2005 \| 10512059 \| 5288 \| 93 \| 21 \| 538 \| 372 \| 548 \| 118 \| 602 \| 742 \| 381 \| 0 \| 381 \| 375 \| 165 \| .. \| 0 \| \| 2006 \| 10599355 \| 5061 \| 104 \| 36 \| 581 \| 379 \| 554 \| 94 \| 522 \| 713 \| 432 \| 0 \| 354 \| 265 \| 149 \| .. \| 2 \| \| 2007 \| 10686386 \| 4962 \| 64 \| 29 \| 530 \| 351 \| 513 \| 154 \| 478 \| 695 \| 421 \| 0 \| 289 \| 279 \| 148 \| .. \| 7 \| \| 2008 \| 10772822 \| 4805 \| 72 \| 31 \| 474 \| 319 \| 487 \| 152 \| 489 \| 622 \| 452 \| 0 \| 286 \| 251 \| 155 \| .. \| 1 \| \| 2009 \| 10861220 \| 5005 \| 116 \| 38 \| 520 \| 364 \| 514 \| 150 \| 571 \| 623 \| 432 \| 0 \| 402 \| 182 \| 151 \| .. \| 5 \| \| 2010 \| 10948634 \| 4678 \| 95 \| 54 \| 452 \| 308 \| 526 \| 173 \| 548 \| 581 \| 412 \| 0 \| 318 \| 161 \| 134 \| .. \| 4 \| \| 2011 \| 10906175 \| 4710 \| 115 \| 66 \| 465 \| 359 \| 505 \| 147 \| 550 \| 663 \| 388 \| 0 \| 296 \| 163 \| 118 \| .. \| 5 \| \| 2012 \| 10864284 \| 4457 \| 113 \| 44 \| 477 \| 357 \| 515 \| 136 \| 526 \| 584 \| 371 \| 0 \| 267 \| 157 \| 101 \| .. \| 2 \| \| 2013 \| 10821967 \| 4501 \| 117 \| 46 \| 430 \| 332 \| 507 \| 145 \| 521 \| 624 \| 368 \| 0 \| 260 \| 199 \| 136 \| .. \| 2 \| \| 2014 \| 10779545 \| 4287 \| 100 \| 41 \| 364 \| 265 \| 496 \| 146 \| 512 \| 603 \| 373 \| 0 \| 330 \| 184 \| 128 \| .. \| 7 \| \| 2015 \| 10736219 \| 4126 \| 92 \| 37 \| 357 \| 274 \| 510 \| 143 \| 539 \| 563 \| 358 \| 0 \| 259 \| 171 \| 118 \| .. \| 3 \| \| 2016 \| 10691213 \| 4140 \| 79 \| 36 \| 342 \| 260 \| 463 \| 135 \| 580 \| 546 \| 381 \| 0 \| 310 \| 191 \| 103 \| .. \| 6 \| \| 2017 \| 10643645 \| 4040 \| 94 \| 49 \| 342 \| 266 \| 487 \| 129 \| 525 \| 456 \| 368 \| 0 \| 325 \| 128 \| 106 \| .. \| 4 \| \| 2018 \| 10593323 \| 3808 \| 101 \| 53 \| 278 \| 233 \| 449 \| 100 \| 536 \| 471 \| 350 \| 0 \| 281 \| 133 \| 86 \| .. \| 4 \| \| 2019 \| 10542010 \| 3886 \| 100 \| 45 \| 260 \| 199 \| 465 \| 111 \| 566 \| 411 \| 371 \| 0 \| 325 \| 141 \| 73 \| .. \| 2 \| \| 2020 \| 10483400 \| 3304 \| 101 \| 47 \| 259 \| 204 \| 449 \| 95 \| 495 \| 356 \| 306 \| 0 \| 274 \| 147 \| 68 \| 96 \| 2 \| \| 2021 \| 10415225 \| 3529 \| 99 \| 45 \| 264 \| 225 \| 416 \| 122 \| 491 \| 361 \| 288 \| 0 \| 202 \| 147 \| 60 \| 95 \| 2 \| \| 2022 \| 10368702 \| 3603 \| 87 \| 35 \| 228 \| 191 \| 427 \| 97 \| 507 \| 380 \| 359 \| 6 \| 328 \| 120 \| 61 \| 85 \| 2 \| |  |  |
| --- | --- | --- | --- | --- | --- | --- | --- | --- | --- | --- | --- | --- | --- | --- | --- | --- | --- | --- | --- | --- | --- | --- | --- | --- | --- | --- | --- | --- | --- | --- | --- | --- | --- | --- | --- | --- | --- | --- | --- | --- | --- | --- | --- | --- | --- | --- | --- | --- | --- | --- | --- | --- | --- | --- | --- | --- | --- | --- | --- | --- | --- | --- | --- | --- | --- | --- | --- | --- | --- | --- | --- | --- | --- | --- | --- | --- | --- | --- | --- | --- | --- | --- | --- | --- | --- | --- | --- | --- | --- | --- | --- | --- | --- | --- | --- | --- | --- | --- | --- | --- | --- | --- | --- | --- | --- | --- | --- | --- | --- | --- | --- | --- | --- | --- | --- | --- | --- | --- | --- | --- | --- | --- | --- | --- | --- | --- | --- | --- | --- | --- | --- | --- | --- | --- | --- | --- | --- | --- | --- | --- | --- | --- | --- | --- | --- | --- | --- | --- | --- | --- | --- | --- | --- | --- | --- | --- | --- | --- | --- | --- | --- | --- | --- | --- | --- | --- | --- | --- | --- | --- | --- | --- | --- | --- | --- | --- | --- | --- | --- | --- | --- | --- | --- | --- | --- | --- | --- | --- | --- | --- | --- | --- | --- | --- | --- | --- | --- | --- | --- | --- | --- | --- | --- | --- | --- | --- | --- | --- | --- | --- | --- | --- | --- | --- | --- | --- | --- | --- | --- | --- | --- | --- | --- | --- | --- | --- | --- | --- | --- | --- | --- | --- | --- | --- | --- | --- | --- | --- | --- | --- | --- | --- | --- | --- | --- | --- | --- | --- | --- | --- | --- | --- | --- | --- | --- | --- | --- | --- | --- | --- | --- | --- | --- | --- | --- | --- | --- | --- | --- | --- | --- | --- | --- | --- | --- | --- | --- | --- | --- | --- | --- | --- | --- | --- | --- | --- | --- | --- | --- | --- | --- | --- | --- | --- | --- | --- | --- | --- | --- | --- | --- | --- | --- | --- | --- | --- | --- | --- | --- | --- | --- | --- | --- | --- | --- | --- | --- | --- | --- | --- | --- | --- | --- | --- | --- | --- | --- | --- | --- | --- | --- | --- | --- | --- | --- | --- | --- | --- | --- | --- | --- | --- | --- | --- | --- | --- | --- | --- | --- | --- | --- | --- | --- | --- | --- | --- | --- | --- | --- | --- | --- | --- | --- | --- | --- | --- | --- | --- | --- | --- | --- | --- | --- | --- | --- | --- | --- | --- | --- | --- | --- | --- | --- | --- | --- | --- | --- | --- | --- | --- | --- | --- | --- | --- | --- | --- | --- | --- | --- | --- | --- | --- | --- | --- | --- | --- | --- | --- | --- | --- | --- | --- | --- | --- | --- | --- | --- | --- | --- | --- | --- | --- | --- | --- | --- | --- | --- | --- | --- | --- | --- | --- | --- | --- |

| Year | Population | Total Deaths | Homicides | Firearm-related injuries | Transport accidents | Motor vehicle crash | Malignant neoplasm | Cardiovascular | Congenital anomalies | Non transport accidents | Neurological disorder | Intentional self-harm | Lower respiratory infection | Acute gastroenteritis | Malnutrition | COVID-19 | Drug Overdose |
| --- | --- | --- | --- | --- | --- | --- | --- | --- | --- | --- | --- | --- | --- | --- | --- | --- | --- |
| 2000 | 10770921 | 4668 | 68 | 20 | 337 | 223 | 455 | 114 | 561 | 535 | 342 | 1 | 371 | 354 | 237 | .. | 4 |
| 2001 | 10653069 | 4387 | 90 | 26 | 369 | 232 | 405 | 93 | 545 | 556 | 328 | 1 | 325 | 304 | 179 | .. | 8 |
| 2002 | 10535365 | 4364 | 75 | 20 | 402 | 282 | 379 | 101 | 542 | 482 | 363 | 1 | 388 | 334 | 231 | .. | 4 |
| 2003 | 10417556 | 4364 | 78 | 20 | 384 | 268 | 420 | 98 | 541 | 438 | 367 | 0 | 325 | 335 | 207 | .. | 6 |
| 2004 | 10299721 | 4345 | 71 | 16 | 377 | 261 | 429 | 105 | 561 | 434 | 331 | 0 | 395 | 323 | 157 | .. | 6 |
| 2005 | 10181409 | 4353 | 74 | 19 | 362 | 257 | 461 | 89 | 577 | 429 | 354 | 0 | 309 | 326 | 169 | .. | 5 |
| 2006 | 10269845 | 4025 | 67 | 13 | 407 | 313 | 405 | 114 | 522 | 468 | 311 | 0 | 286 | 245 | 143 | .. | 3 |
| 2007 | 10358179 | 4096 | 55 | 25 | 337 | 250 | 454 | 125 | 507 | 418 | 348 | 0 | 265 | 251 | 165 | .. | 3 |
| 2008 | 10446104 | 3830 | 59 | 18 | 351 | 277 | 410 | 145 | 472 | 381 | 299 | 0 | 234 | 249 | 144 | .. | 4 |
| 2009 | 10535024 | 4143 | 89 | 23 | 384 | 280 | 422 | 139 | 519 | 392 | 352 | 0 | 371 | 184 | 155 | .. | 0 |
| 2010 | 10623437 | 3852 | 82 | 38 | 355 | 259 | 442 | 122 | 526 | 369 | 320 | 0 | 255 | 148 | 139 | .. | 4 |
| 2011 | 10588941 | 3809 | 74 | 36 | 332 | 257 | 451 | 128 | 522 | 414 | 302 | 0 | 258 | 169 | 126 | .. | 6 |
| 2012 | 10554599 | 3709 | 74 | 25 | 325 | 254 | 374 | 142 | 535 | 374 | 332 | 0 | 242 | 166 | 107 | .. | 1 |
| 2013 | 10520096 | 3505 | 76 | 20 | 245 | 205 | 384 | 150 | 487 | 327 | 327 | 0 | 236 | 152 | 130 | .. | 4 |
| 2014 | 10485542 | 3485 | 74 | 18 | 245 | 189 | 395 | 130 | 530 | 366 | 293 | 0 | 262 | 139 | 124 | .. | 4 |
| 2015 | 10450564 | 3382 | 77 | 24 | 269 | 215 | 393 | 128 | 498 | 357 | 289 | 0 | 245 | 140 | 94 | .. | 5 |
| 2016 | 10414713 | 3462 | 67 | 22 | 255 | 203 | 413 | 97 | 554 | 323 | 281 | 0 | 321 | 172 | 91 | .. | 3 |
| 2017 | 10377669 | 3442 | 72 | 25 | 226 | 196 | 393 | 104 | 545 | 343 | 301 | 0 | 336 | 122 | 105 | .. | 2 |
| 2018 | 10338942 | 3265 | 93 | 39 | 215 | 175 | 386 | 117 | 536 | 286 | 271 | 0 | 259 | 101 | 85 | .. | 2 |
| 2019 | 10299927 | 3304 | 83 | 41 | 188 | 158 | 370 | 96 | 561 | 275 | 299 | 0 | 331 | 141 | 96 | .. | 4 |
| 2020 | 10257435 | 2646 | 85 | 38 | 144 | 121 | 337 | 80 | 382 | 207 | 241 | 0 | 216 | 95 | 35 | 76 | 2 |
| 2021 | 10205637 | 2854 | 63 | 23 | 185 | 157 | 337 | 83 | 485 | 237 | 250 | 0 | 195 | 105 | 70 | 91 | 0 |
| 2022 | 10184235 | 2944 | 61 | 29 | 176 | 150 | 330 | 85 | 504 | 199 | 275 | 2 | 272 | 119 | 66 | 72 | 2 |

**Supplementary Table 4. Population of 1-9 years old female children and deaths by cause from 2000 to 2022 in Mexico**

**Supplementary Table 5. Population of 10-19 years old male adolescents and deaths by cause from 2000 to 2022 in Mexico**

|  |  |  |  |  |
| --- | --- | --- | --- | --- |
| \| Year \| Population \| Total Deaths \| Homicides \| Firearm-related injuries \| Transport accidents \| Motor vehicle crash \| Malignant neoplasm \| Cardiovascular \| Congenital anomalies \| Non transport accidents \| Neurological disorder \| Intentional self-harm \| Lower respiratory infection \| Acute gastroenteritis \| Malnutrition \| COVID-19 \| Drug Overdose \| \| --- \| --- \| --- \| --- \| --- \| --- \| --- \| --- \| --- \| --- \| --- \| --- \| --- \| --- \| --- \| --- \| --- \| --- \| \| 2000 \| 10345385 \| 7358 \| 979 \| 705 \| 1487 \| 1064 \| 621 \| 232 \| 174 \| 1038 \| 382 \| 410 \| 114 \| 37 \| 57 \| .. \| 9 \| \| 2001 \| 10306099 \| 7413 \| 974 \| 692 \| 1452 \| 1060 \| 625 \| 214 \| 172 \| 1072 \| 400 \| 450 \| 94 \| 41 \| 54 \| .. \| 19 \| \| 2002 \| 10266813 \| 7286 \| 921 \| 658 \| 1582 \| 1208 \| 646 \| 264 \| 218 \| 1050 \| 423 \| 458 \| 103 \| 41 \| 52 \| .. \| 13 \| \| 2003 \| 10227527 \| 7286 \| 812 \| 589 \| 1554 \| 1204 \| 686 \| 226 \| 192 \| 984 \| 468 \| 468 \| 95 \| 34 \| 75 \| .. \| 11 \| \| 2004 \| 10188241 \| 7185 \| 732 \| 533 \| 1597 \| 1277 \| 676 \| 252 \| 219 \| 856 \| 443 \| 488 \| 110 \| 30 \| 54 \| .. \| 17 \| \| 2005 \| 10541816 \| 7287 \| 789 \| 531 \| 1588 \| 1268 \| 666 \| 267 \| 224 \| 877 \| 509 \| 472 \| 101 \| 27 \| 54 \| .. \| 13 \| \| 2006 \| 10647000 \| 7488 \| 795 \| 543 \| 1703 \| 1388 \| 714 \| 301 \| 229 \| 889 \| 458 \| 486 \| 106 \| 39 \| 48 \| .. \| 7 \| \| 2007 \| 10752184 \| 7660 \| 664 \| 515 \| 1576 \| 1304 \| 685 \| 321 \| 195 \| 1068 \| 500 \| 464 \| 108 \| 36 \| 52 \| .. \| 5 \| \| 2008 \| 10857368 \| 8167 \| 1044 \| 833 \| 1807 \| 1483 \| 704 \| 346 \| 239 \| 1047 \| 462 \| 532 \| 103 \| 33 \| 59 \| .. \| 6 \| \| 2009 \| 10962552 \| 8824 \| 1497 \| 1184 \| 1832 \| 1547 \| 689 \| 355 \| 256 \| 963 \| 511 \| 566 \| 176 \| 32 \| 72 \| .. \| 9 \| \| 2010 \| 11067734 \| 9226 \| 2069 \| 1712 \| 1635 \| 1374 \| 712 \| 365 \| 253 \| 964 \| 491 \| 584 \| 109 \| 39 \| 54 \| .. \| 7 \| \| 2011 \| 11062602 \| 9240 \| 2292 \| 1905 \| 1576 \| 1362 \| 703 \| 342 \| 260 \| 823 \| 505 \| 740 \| 103 \| 36 \| 75 \| .. \| 10 \| \| 2012 \| 11057470 \| 9081 \| 2222 \| 1787 \| 1566 \| 1319 \| 750 \| 353 \| 231 \| 884 \| 481 \| 714 \| 125 \| 27 \| 63 \| .. \| 15 \| \| 2013 \| 11052338 \| 8089 \| 1683 \| 1267 \| 1420 \| 1195 \| 712 \| 373 \| 224 \| 812 \| 502 \| 642 \| 115 \| 26 \| 52 \| .. \| 10 \| \| 2014 \| 11047206 \| 7668 \| 1309 \| 983 \| 1403 \| 1192 \| 814 \| 373 \| 239 \| 766 \| 486 \| 647 \| 118 \| 32 \| 65 \| .. \| 10 \| \| 2015 \| 11042074 \| 7844 \| 1413 \| 1050 \| 1369 \| 1160 \| 813 \| 384 \| 185 \| 795 \| 506 \| 717 \| 114 \| 45 \| 51 \| .. \| 10 \| \| 2016 \| 11036942 \| 7977 \| 1632 \| 1220 \| 1430 \| 1228 \| 776 \| 336 \| 245 \| 740 \| 501 \| 648 \| 131 \| 37 \| 63 \| .. \| 9 \| \| 2017 \| 11031810 \| 8735 \| 2226 \| 1763 \| 1443 \| 1276 \| 777 \| 377 \| 229 \| 676 \| 524 \| 682 \| 138 \| 26 \| 67 \| .. \| 17 \| \| 2018 \| 11026678 \| 8687 \| 2321 \| 1835 \| 1406 \| 1247 \| 781 \| 361 \| 233 \| 635 \| 545 \| 686 \| 123 \| 38 \| 70 \| .. \| 10 \| \| 2019 \| 11021546 \| 8444 \| 2222 \| 1852 \| 1250 \| 1131 \| 803 \| 335 \| 250 \| 606 \| 516 \| 693 \| 128 \| 41 \| 63 \| .. \| 15 \| \| 2020 \| 11016410 \| 8667 \| 2189 \| 1816 \| 1297 \| 1166 \| 793 \| 359 \| 265 \| 605 \| 471 \| 745 \| 182 \| 34 \| 45 \| 169 \| 14 \| \| 2021 \| 11021542 \| 8879 \| 2086 \| 1697 \| 1367 \| 1245 \| 785 \| 401 \| 257 \| 593 \| 501 \| 694 \| 130 \| 40 \| 56 \| 290 \| 16 \| \| 2022 \| 11016410 \| 8865 \| 2063 \| 1695 \| 1442 \| 1340 \| 768 \| 363 \| 266 \| 605 \| 564 \| 690 \| 145 \| 35 \| 54 \| 96 \| 16 \| | | |  |  |

**Supplementary Table 6. Population of 10-19 years old female adolescents and deaths by cause from 2000 to 2022 in Mexico**

| Year | Population aged 1-19 | Total Deaths | Homicides | Firearm-related injuries | Transport accidents | Motor vehicle crash | Malignant neoplasm | Cardiovascular | Congenital anomalies | Non transport accidents | Neurological disorder | Intentional self-harm | Lower respiratory infection | Acute gastroenteritis | Malnutrition | COVID-19 | Drug Overdose |
| --- | --- | --- | --- | --- | --- | --- | --- | --- | --- | --- | --- | --- | --- | --- | --- | --- | --- |
| 2000 | 10383243 | 3748 | 207 | 127 | 565 | 386 | 689 | 238 | 325 | 405 | 406 | 169 | 141 | 102 | 102 | .. | 28 |
| 2001 | 10410460 | 3820 | 236 | 144 | 684 | 486 | 681 | 249 | 299 | 434 | 403 | 220 | 132 | 80 | 84 | .. | 23 |
| 2002 | 10437677 | 3845 | 209 | 110 | 652 | 499 | 695 | 229 | 309 | 365 | 462 | 222 | 157 | 80 | 91 | .. | 25 |
| 2003 | 10464894 | 3845 | 230 | 111 | 682 | 526 | 638 | 255 | 351 | 362 | 430 | 219 | 127 | 78 | 95 | .. | 26 |
| 2004 | 10492111 | 3701 | 209 | 94 | 663 | 520 | 710 | 240 | 316 | 383 | 430 | 189 | 127 | 70 | 91 | .. | 23 |
| 2005 | 10519328 | 3854 | 230 | 107 | 691 | 558 | 732 | 222 | 317 | 363 | 486 | 218 | 135 | 78 | 88 | .. | 9 |
| 2006 | 10595125 | 3862 | 222 | 106 | 669 | 544 | 706 | 230 | 317 | 348 | 418 | 236 | 115 | 68 | 61 | .. | 17 |
| 2007 | 10670922 | 3907 | 178 | 114 | 648 | 539 | 721 | 276 | 303 | 398 | 467 | 194 | 108 | 67 | 80 | .. | 15 |
| 2008 | 10746719 | 4020 | 219 | 112 | 668 | 549 | 748 | 261 | 290 | 353 | 410 | 247 | 114 | 65 | 92 | .. | 19 |
| 2009 | 10822516 | 4414 | 286 | 152 | 692 | 573 | 728 | 326 | 312 | 356 | 506 | 326 | 299 | 60 | 90 | .. | 17 |
| 2010 | 10898315 | 4283 | 381 | 232 | 672 | 543 | 767 | 315 | 292 | 346 | 456 | 284 | 112 | 55 | 102 | .. | 20 |
| 2011 | 10881865 | 4200 | 398 | 221 | 587 | 470 | 773 | 290 | 255 | 346 | 446 | 366 | 130 | 73 | 84 | .. | 24 |
| 2012 | 10865415 | 4152 | 461 | 251 | 637 | 557 | 656 | 288 | 292 | 291 | 486 | 315 | 136 | 55 | 62 | .. | 19 |
| 2013 | 10848965 | 3781 | 351 | 183 | 485 | 420 | 731 | 278 | 294 | 259 | 410 | 308 | 115 | 48 | 79 | .. | 16 |
| 2014 | 10832515 | 3878 | 349 | 139 | 502 | 416 | 723 | 351 | 296 | 308 | 433 | 356 | 142 | 53 | 86 | .. | 22 |
| 2015 | 10816065 | 3734 | 317 | 168 | 495 | 428 | 718 | 289 | 283 | 305 | 431 | 374 | 119 | 42 | 83 | .. | 14 |
| 2016 | 10799615 | 3794 | 358 | 210 | 521 | 429 | 721 | 266 | 293 | 288 | 441 | 353 | 167 | 45 | 76 | .. | 10 |
| 2017 | 10783165 | 3945 | 436 | 235 | 500 | 432 | 808 | 242 | 348 | 284 | 461 | 315 | 173 | 42 | 74 | .. | 14 |
| 2018 | 10766715 | 3806 | 474 | 293 | 420 | 367 | 734 | 253 | 343 | 230 | 456 | 341 | 143 | 40 | 92 | .. | 18 |
| 2019 | 10750265 | 3835 | 464 | 285 | 416 | 371 | 734 | 281 | 319 | 241 | 460 | 332 | 158 | 54 | 79 | .. | 14 |
| 2020 | 10733820 | 3922 | 430 | 290 | 332 | 293 | 732 | 261 | 306 | 194 | 372 | 413 | 165 | 43 | 47 | 192 | 10 |
| 2021 | 10717370 | 4096 | 461 | 288 | 390 | 353 | 699 | 281 | 287 | 232 | 425 | 401 | 126 | 37 | 59 | 309 | 12 |
| 2022 | 10700921 | 4123 | 407 | 295 | 394 | 367 | 507 | 230 | 226 | 174 | 338 | 399 | 101 | 17 | 38 | 106 | 13 |

**Supplementary Table 7. Population of children and adolescents 1-19 years old and deaths by cause from 2000 to 2022 in the US**

|  | Population | Total Deaths | Homicides | Firearm-related injuries | Transport accidents | Motor vehicle crash | Malignant neoplasm | Cardiovascular | Congenital anomalies | Non- transport accidents | Neurological disorder | Intentional self-harm | Lower respiratory infection | Acute gastroenteritis | Malnutrition | COVID-19 |
| --- | --- | --- | --- | --- | --- | --- | --- | --- | --- | --- | --- | --- | --- | --- | --- | --- |
| 2000 | 76,667,617 | 25,955 | 2,641 | 2,999 | 8,088 | 7,674 | 2,179 | 818 | 1,119 | 3,472 | 1,196 | 1,928 | 277 | 13 | 0 | .. |
| 2001 | 76,893,883 | 25,757 | 2,640 | 2,900 | 7,894 | 7,520 | 2,160 | 843 | 1,188 | 3,302 | 1,211 | 1,890 | 291 | 24 | 0 | .. |
| 2002 | 77,221,939 | 25,820 | 2,671 | 2,857 | 8,160 | 7,858 | 2,197 | 829 | 1,195 | 3,336 | 1,183 | 1,777 | 308 | 17 | 0 | .. |
| 2003 | 77,449,945 | 25,514 | 2,638 | 2,819 | 7,885 | 7,532 | 2,158 | 876 | 1,150 | 3,205 | 1,226 | 1,737 | 425 | 13 | 0 | .. |
| 2004 | 77,740,096 | 25,325 | 2,638 | 2,818 | 7,808 | 7,512 | 2,149 | 777 | 1,215 | 3,324 | 1,239 | 1,985 | 288 | 18 | 0 | .. |
| 2005 | 78,000,867 | 25,061 | 2,792 | 2,999 | 7,321 | 7,031 | 2,108 | 846 | 1,171 | 3,374 | 1,235 | 1,885 | 305 | 28 | 0 | .. |
| 2006 | 78,282,680 | 24,519 | 3,047 | 3,178 | 7,160 | 6,866 | 1,959 | 819 | 1,087 | 3,367 | 1,173 | 1,774 | 284 | 19 | 0 | .. |
| 2007 | 78,601,434 | 24,149 | 2,968 | 3,026 | 6,811 | 6,559 | 1,996 | 768 | 1,111 | 3,464 | 1,196 | 1,665 | 285 | 24 | 0 | .. |
| 2008 | 78,985,529 | 22,788 | 2,836 | 2,938 | 5,582 | 5,348 | 1,969 | 824 | 1,082 | 3,287 | 1,180 | 1,826 | 321 | 13 | 0 | .. |
| 2009 | 79,276,804 | 21,621 | 2,600 | 2,780 | 5,029 | 4,818 | 1,890 | 683 | 1,058 | 2,933 | 1,140 | 1,934 | 564 | 30 | 10 | .. |
| 2010 | 79,323,403 | 20,482 | 2,478 | 2,683 | 4,551 | 4,340 | 1,863 | 730 | 1,007 | 3,023 | 1,073 | 1,933 | 227 | 39 | 0 | .. |
| 2011 | 78,848,612 | 20,241 | 2,375 | 2,663 | 4,373 | 4,169 | 1,852 | 706 | 1,058 | 2,937 | 1,061 | 2,089 | 318 | 36 | 0 | .. |
| 2012 | 78,561,723 | 19,492 | 2,264 | 2,647 | 4,185 | 3,981 | 1,932 | 669 | 1,029 | 2,713 | 1,036 | 2,093 | 252 | 38 | 0 | .. |
| 2013 | 78,306,304 | 18,888 | 2,021 | 2,430 | 3,858 | 3,685 | 1,850 | 628 | 982 | 2,631 | 1,069 | 2,143 | 317 | 38 | 0 | .. |
| 2014 | 78,187,252 | 18,666 | 2,049 | 2,518 | 3,909 | 3,749 | 1,785 | 609 | 926 | 2,511 | 997 | 2,265 | 296 | 42 | 0 | .. |
| 2015 | 78,147,652 | 19,562 | 2,254 | 2,790 | 4,084 | 3,896 | 1,802 | 644 | 967 | 2,588 | 1,107 | 2,474 | 267 | 45 | 0 | .. |
| 2016 | 78,134,923 | 20,360 | 2,441 | 3,116 | 4,254 | 4,056 | 1,853 | 618 | 979 | 2,793 | 1,031 | 2,560 | 265 | 39 | 0 | .. |
| 2017 | 78,213,917 | 20,337 | 2,470 | 3,397 | 4,142 | 3,947 | 1,740 | 674 | 971 | 2,651 | 1,023 | 3,013 | 314 | 34 | 0 | .. |
| 2018 | 78,134,457 | 19,660 | 2,397 | 3,309 | 3,634 | 3,496 | 1,790 | 596 | 929 | 2,566 | 1,074 | 3,009 | 345 | 35 | 0 | .. |
| 2019 | 77,842,364 | 19,431 | 2,507 | 3,359 | 3,643 | 3,486 | 1,649 | 609 | 985 | 2,535 | 1,006 | 2,756 | 341 | 31 | 0 | .. |
| 2020 | 77,519,345 | 21,430 | 3,337 | 4,332 | 4,044 | 3,879 | 1,648 | 636 | 887 | 3,329 | 975 | 2,817 | 274 | 21 | 13 | 186 |
| 2021 | 78,566843 | 23,198 | 3,553 | 4,720 | 4,433 | 4,290 | 1,670 | 699 | 964 | 3692 | 1,099 | 2950 | 157 | 34 | 12 | 547 |
| 2022 | 77,390366 | 23,140 | 3,621 | 4,584 | 4,115 | 3,967 | 1,732 | 659 | 1,103 | 3,587 | 1,130 | 2,664 | 353 | 48 | 17 | 393 |

**Supplementary Table 8. Population of 1-9 years old female children and deaths by cause from 2000 to 2022 in the US**

| Year | Population | Total Deaths | Homicides | Firearm-related injuries | Transport accidents | Motor vehicle crash | Malignant neoplasm | Cardiovascular | Congenital anomalies | Non transport accidents | Neurological disorder | Intentional self-harm | Lower respiratory infection | Acute gastroenteritis | Malnutrition | COVID-19 |  |
| --- | --- | --- | --- | --- | --- | --- | --- | --- | --- | --- | --- | --- | --- | --- | --- | --- | --- |
| 2000 | 17,534,662 | 3,558 | 221 | 42 | 678 | 634 | 407 | 65 | 329 | 617 | 262 | 0 | 83 | 12 | 0 | .. |  |
| 2001 | 17,312,834 | 3,574 | 239 | 59 | 608 | 570 | 431 | 93 | 356 | 558 | 259 | 0 | 89 | 7 | 0 | .. |  |
| 2002 | 17,264,446 | 3,368 | 265 | 56 | 559 | 528 | 426 | 63 | 348 | 514 | 226 | 0 | 84 | 10 | 0 | .. |  |
| 2003 | 17,217,940 | 3,397 | 226 | 43 | 576 | 541 | 388 | 50 | 352 | 539 | 245 | 0 | 122 | 0 | 0 | .. |  |
| 2004 | 17,216,406 | 3,379 | 233 | 46 | 589 | 562 | 410 | 78 | 345 | 532 | 257 | 0 | 93 | 16 | 0 | .. |  |
| 2005 | 17,259,023 | 3,272 | 214 | 42 | 561 | 540 | 382 | 61 | 368 | 521 | 224 | 0 | 86 | 18 | 0 | .. |  |
| 2006 | 17,330,265 | 3,274 | 229 | 57 | 553 | 525 | 371 | 42 | 363 | 556 | 201 | 0 | 100 | 16 | 0 | .. |  |
| 2007 | 17,453,771 | 3,261 | 252 | 53 | 489 | 464 | 398 | 85 | 359 | 505 | 216 | 0 | 95 | 9 | 0 | .. |  |
| 2008 | 17,639,782 | 3,143 | 238 | 50 | 401 | 384 | 407 | 85 | 325 | 493 | 205 | 0 | 96 | 0 | 0 | .. |  |
| 2009 | 17,818,004 | 3,080 | 246 | 54 | 408 | 387 | 382 | 80 | 322 | 446 | 222 | 0 | 142 | 11 | 0 | .. |  |
| 2010 | 17,911,077 | 2,861 | 203 | 46 | 361 | 345 | 349 | 67 | 358 | 440 | 191 | 0 | 78 | 12 | 0 | .. |  |
| 2011 | 17,859,740 | 2,912 | 227 | 56 | 395 | 375 | 367 | 77 | 300 | 443 | 192 | 0 | 106 | 16 | 0 | .. |  |
| 2012 | 17,873,213 | 2,837 | 205 | 59 | 386 | 362 | 360 | 63 | 310 | 447 | 199 | 0 | 65 | 10 | 0 | .. |  |
| 2013 | 17,852,221 | 2,789 | 205 | 45 | 368 | 349 | 351 | 47 | 326 | 413 | 208 | 0 | 93 | 11 | 0 | .. |  |
| 2014 | 17,831,799 | 2,658 | 230 | 62 | 349 | 343 | 333 | 36 | 283 | 389 | 171 | 0 | 90 | 11 | 0 | .. |  |
| 2015 | 17,814,820 | 2,709 | 194 | 58 | 375 | 359 | 353 | 59 | 284 | 406 | 197 | 0 | 69 | 17 | 0 | .. |  |
| 2016 | 17,800,393 | 2,902 | 225 | 74 | 390 | 378 | 367 | 41 | 331 | 413 | 203 | 0 | 76 | 16 | 0 | .. |  |
| 2017 | 17,754,844 | 2,703 | 206 | 62 | 396 | 376 | 360 | 36 | 283 | 398 | 174 | 0 | 81 | 6 | 0 | .. |  |
| 2018 | 17,678,022 | 2,631 | 195 | 61 | 342 | 324 | 332 | 39 | 281 | 416 | 171 | 0 | 100 | 21 | 0 | .. |  |
| 2019 | 17,592,674 | 2,678 | 180 | 46 | 363 | 347 | 313 | 53 | 293 | 411 | 169 | 12 | 94 | 10 | 0 | .. |  |
| 2020 | 17,504,224 | 2,468 | 211 | 78 | 322 | 307 | 327 | 41 | 268 | 370 | 156 | 7 | 69 | 4 | 0 | 15 |  |
| 2021 | 17,375,385 | 2,742 | 206 | 91 | 370 | 356 | 286 | 61 | 301 | 488 | 182 | 0 | 46 | 3 | 0 | 50 |  |
| 2022 | 17,040261 | 2947 | 219 | 83 | 330 | 319 | 319 | 31 | 341 | 468 | 168 | 0 | 102 | 17 | 0 | 70 |  |

**Supplementary Table 9. Population of 1-9 years old male children and deaths by cause from 2000 to 2022 in the US**

|  | Population | Total Deaths | Homicides | Firearm-related injuries | Transport accidents | Motor vehicle crash | Malignant neoplasm | Cardiovascular | Congenital anomalies | Non- transport accidents | Neurological disorder | Intentional self-harm | Lower respiratory infection | Acute gastroenteritis | Malnutrition | COVID-19 |
| --- | --- | --- | --- | --- | --- | --- | --- | --- | --- | --- | --- | --- | --- | --- | --- | --- |
| 2000 | 18384993 | 4674 | 275 | 72 | 855 | 797 | 502 | 95 | 364 | 1067 | 281 | 0 | 81 | 0 | 0 | .. |
| 2001 | 18146087 | 4626 | 313 | 88 | 839 | 774 | 482 | 106 | 383 | 992 | 285 | 0 | 90 | 14 | 0 | .. |
| 2002 | 18085702 | 4508 | 298 | 73 | 786 | 746 | 513 | 57 | 381 | 958 | 318 | 0 | 94 | 0 | 0 | .. |
| 2003 | 18019486 | 4466 | 272 | 65 | 767 | 726 | 520 | 64 | 369 | 931 | 286 | 0 | 147 | 0 | 0 | .. |
| 2004 | 18009458 | 4294 | 266 | 62 | 751 | 713 | 515 | 41 | 429 | 895 | 286 | 0 | 76 | 0 | 0 | .. |
| 2005 | 18043051 | 4321 | 282 | 76 | 734 | 700 | 480 | 66 | 350 | 920 | 290 | 0 | 95 | 0 | 0 | .. |
| 2006 | 18111568 | 4092 | 286 | 78 | 679 | 640 | 465 | 84 | 334 | 866 | 260 | 0 | 80 | 0 | 0 | .. |
| 2007 | 18238805 | 4153 | 279 | 80 | 644 | 602 | 446 | 62 | 383 | 915 | 265 | 0 | 80 | 12 | 0 | .. |
| 2008 | 18428212 | 4089 | 296 | 83 | 544 | 513 | 444 | 69 | 366 | 866 | 261 | 0 | 111 | 0 | 0 | .. |
| 2009 | 18605426 | 3893 | 249 | 81 | 550 | 515 | 445 | 67 | 337 | 835 | 254 | 0 | 133 | 15 | 0 | .. |
| 2010 | 18694789 | 3785 | 293 | 91 | 526 | 499 | 436 | 77 | 312 | 825 | 258 | 0 | 65 | 14 | 0 | .. |
| 2011 | 18639977 | 3785 | 314 | 88 | 474 | 444 | 427 | 70 | 375 | 826 | 231 | 0 | 87 | 11 | 0 | .. |
| 2012 | 18658590 | 3715 | 272 | 83 | 496 | 470 | 472 | 49 | 358 | 767 | 235 | 0 | 93 | 18 | 0 | .. |
| 2013 | 18644665 | 3706 | 257 | 84 | 484 | 460 | 424 | 74 | 329 | 797 | 245 | 0 | 90 | 11 | 0 | .. |
| 2014 | 18616300 | 3529 | 257 | 79 | 480 | 452 | 424 | 48 | 308 | 728 | 229 | 0 | 96 | 13 | 0 | .. |
| 2015 | 18601599 | 3658 | 315 | 93 | 485 | 453 | 438 | 66 | 332 | 724 | 248 | 0 | 85 | 14 | 0 | .. |
| 2016 | 18586298 | 3,633 | 253 | 108 | 489 | 466 | 459 | 61 | 305 | 756 | 206 | 0 | 94 | 13 | 0 | .. |
| 2017 | 18548959 | 3531 | 251 | 116 | 460 | 437 | 383 | 62 | 329 | 731 | 232 | 0 | 113 | 15 | 0 | .. |
| 2018 | 18479687 | 3529 | 279 | 90 | 430 | 406 | 387 | 37 | 304 | 772 | 237 | 0 | 119 | 0 | 0 | .. |
| 2019 | 18396852 | 3331 | 259 | 102 | 414 | 388 | 343 | 52 | 315 | 675 | 212 | 0 | 102 | 12 | 0 | .. |
| 2020 | 18299769 | 3271 | 269 | 168 | 427 | 408 | 362 | 36 | 285 | 719 | 197 | 13 | 90 | 12 | 0 | 21 |
| 2021 | 18179008 | 3480 | 291 | 180 | 464 | 448 | 343 | 50 | 282 | 804 | 195 | 0 | 46 | 16 | 0 | 67 |
| 2022 | 17824174 | 3776 | 304 | 164 | 434 | 410 | 340 | 59 | 341 | 782 | 242 | 0 | 121 | 18 | 0 | 93 |

| Year | Population | Total Deaths | Homicides | Firearm-related injuries | Transport accidents | Motor vehicle crash | Malignant neoplasm | Cardiovascular | Congenital anomalies | Non transport accidents | Neurological disorder | Intentional self-harm | Lower respiratory infection | Acute gastroenteritis | Malnutrition | COVID-19 | Drug Overdose |
| --- | --- | --- | --- | --- | --- | --- | --- | --- | --- | --- | --- | --- | --- | --- | --- | --- | --- |
| 2000 | 20911201 | 12248 | 1763 | 3255 | 4367 | 4141 | 746 | 251 | 239 | 1427 | 399 | 1589 | 56 | 0 | 0 | .. | 296 |
| 2001 | 21294596 | 12207 | 1750 | 3183 | 4362 | 4157 | 727 | 260 | 270 | 1377 | 435 | 1552 | 60 | 0 | 0 | .. | 350 |
| 2002 | 21518490 | 12340 | 1737 | 3128 | 4509 | 4343 | 727 | 310 | 262 | 1465 | 405 | 1476 | 64 | 0 | 0 | .. | 426 |
| 2003 | 21681077 | 12216 | 1822 | 3116 | 4336 | 4136 | 736 | 263 | 248 | 1382 | 449 | 1410 | 83 | 0 | 0 | .. | 460 |
| 2004 | 21827733 | 12032 | 1769 | 3164 | 4224 | 4059 | 704 | 246 | 245 | 1499 | 458 | 1530 | 70 | 0 | 0 | .. | 557 |
| 2005 | 21915622 | 12183 | 1951 | 3342 | 3977 | 3808 | 736 | 253 | 281 | 1534 | 472 | 1505 | 77 | 0 | 0 | .. | 546 |
| 2006 | 21981610 | 11992 | 2158 | 3455 | 3913 | 3745 | 665 | 266 | 231 | 1551 | 453 | 1413 | 54 | 0 | 0 | .. | 628 |
| 2007 | 22007450 | 11624 | 2076 | 3303 | 3733 | 3609 | 639 | 237 | 210 | 1583 | 452 | 1349 | 50 | 0 | 0 | .. | 726 |
| 2008 | 22003913 | 10842 | 1942 | 3236 | 3090 | 2954 | 638 | 262 | 226 | 1534 | 445 | 1433 | 54 | 0 | 0 | .. | 693 |
| 2009 | 21960071 | 10026 | 1782 | 3132 | 2637 | 2518 | 597 | 196 | 226 | 1295 | 395 | 1496 | 142 | 0 | 0 | .. | 591 |
| 2010 | 21883528 | 9595 | 1685 | 3084 | 2413 | 2290 | 603 | 252 | 194 | 1360 | 387 | 1503 | 49 | 10 | 0 | .. | 616 |
| 2011 | 21705976 | 9417 | 1548 | 3078 | 2320 | 2218 | 628 | 195 | 219 | 1296 | 405 | 1629 | 71 | 0 | 0 | .. | 605 |
| 2012 | 21530075 | 8940 | 1509 | 3037 | 2158 | 2045 | 638 | 197 | 204 | 1181 | 381 | 1593 | 45 | 0 | 0 | .. | 492 |
| 2013 | 21398713 | 8453 | 1333 | 2865 | 1979 | 1879 | 597 | 181 | 175 | 1075 | 397 | 1595 | 74 | 0 | 0 | .. | 449 |
| 2014 | 21335242 | 8599 | 1315 | 2937 | 2105 | 2006 | 604 | 191 | 190 | 1084 | 372 | 1682 | 59 | 0 | 0 | .. | 425 |
| 2015 | 21318255 | 8963 | 1482 | 3202 | 2145 | 2047 | 573 | 170 | 198 | 1088 | 415 | 1788 | 61 | 10 | 0 | .. | 501 |
| 2016 | 21320578 | 9,477 | 1633 | 3434 | 2206 | 2081 | 605 | 142 | 214 | 1223 | 378 | 1866 | 53 | 0 | 0 | .. | 580 |
| 2017 | 21405563 | 9819 | 1705 | 3965 | 2193 | 2083 | 588 | 192 | 195 | 1126 | 392 | 2286 | 55 | 0 | 0 | .. | 545 |
| 2018 | 21433748 | 9175 | 1600 | 3858 | 1807 | 1734 | 632 | 156 | 197 | 983 | 413 | 2256 | 67 | 0 | 0 | .. | 463 |
| 2019 | 21363868 | 9257 | 1755 | 3801 | 1894 | 1811 | 573 | 146 | 218 | 1080 | 387 | 2030 | 67 | 0 | 0 | .. | 527 |
| 2020 | 21289565 | 11068 | 2445 | 4511 | 2236 | 2143 | 548 | 138 | 180 | 1679 | 386 | 2066 | 58 | 0 | 0 | 77 | 1085 |
| 2021 | 22011199 | 11823 | 1225 | 4743 | 2416 | 2336 | 642 | 232 | 215 | 1746 | 431 | 2137 | 39 | 0 | 0 | 256 | 1151 |
| 2022 | 21783896 | 11344 | 2665 | 4612 | 2269 | 2188 | 593 | 197 | 250 | 1623 | 461 | 1946 | 66 | 10 | 0 | 128 | 1110 |

**Supplementary Table 10. Population of 10-19 years old male adolescents and deaths by cause from 2000 to 2022 in the US**

|  |  |  |  |  |
| --- | --- | --- | --- | --- |
|  | | |  |  |

**Supplementary Table 11. Population of 10-19 years old female adolescents and deaths by cause from 2000 to 2021 in the US**

| Year | Population aged 1-19 | Total Deaths | Homicides | Firearm-related injuries | Transport accidents | Motor vehicle crash | Malignant neoplasm | Cardiovascular | Congenital anomalies | Non transport accidents | Neurological disorder | Intentional self-harm | Lower respiratory infection | Acute gastroenteritis | Malnutrition | COVID-19 | Drug Overdose |
| --- | --- | --- | --- | --- | --- | --- | --- | --- | --- | --- | --- | --- | --- | --- | --- | --- | --- |
| 2000 | 19836761 | 5475 | 382 | 332 | 2188 | 2102 | 524 | 163 | 187 | 361 | 254 | 332 | 49 | 0 | 0 | .. | 104 |
| 2001 | 20140366 | 5350 | 338 | 307 | 2085 | 2019 | 520 | 124 | 179 | 375 | 232 | 331 | 52 | 0 | 0 | .. | 134 |
| 2002 | 20353301 | 5604 | 371 | 245 | 2306 | 2241 | 531 | 115 | 204 | 399 | 234 | 297 | 64 | 0 | 0 | .. | 157 |
| 2003 | 20531442 | 5435 | 318 | 271 | 2206 | 2129 | 514 | 121 | 181 | 353 | 246 | 321 | 69 | 0 | 0 | .. | 163 |
| 2004 | 20686499 | 5620 | 370 | 329 | 2244 | 2178 | 520 | 115 | 196 | 398 | 238 | 453 | 46 | 0 | 0 | .. | 197 |
| 2005 | 20783171 | 5285 | 345 | 306 | 2049 | 1983 | 510 | 102 | 172 | 399 | 249 | 378 | 46 | 0 | 0 | .. | 196 |
| 2006 | 20859237 | 5161 | 374 | 301 | 2015 | 1956 | 458 | 116 | 159 | 394 | 259 | 358 | 47 | 0 | 0 | .. | 211 |
| 2007 | 20901408 | 5111 | 361 | 305 | 1945 | 1884 | 513 | 110 | 159 | 461 | 263 | 312 | 57 | 0 | 0 | .. | 230 |
| 2008 | 20913622 | 4714 | 360 | 306 | 1547 | 1497 | 480 | 132 | 165 | 394 | 269 | 386 | 59 | 0 | 0 | .. | 198 |
| 2009 | 20893303 | 4622 | 323 | 292 | 1434 | 1398 | 466 | 81 | 173 | 357 | 269 | 432 | 143 | 0 | 0 | .. | 198 |
| 2010 | 20834009 | 4241 | 297 | 284 | 1251 | 1206 | 475 | 91 | 143 | 398 | 237 | 423 | 33 | 0 | 0 | .. | 222 |
| 2011 | 20642919 | 4127 | 286 | 308 | 1184 | 1132 | 430 | 81 | 164 | 372 | 233 | 455 | 50 | 0 | 0 | .. | 218 |
| 2012 | 20499845 | 4000 | 278 | 300 | 1145 | 1104 | 462 | 89 | 157 | 318 | 221 | 495 | 43 | 0 | 0 | .. | 165 |
| 2013 | 20410705 | 3940 | 226 | 253 | 1027 | 997 | 478 | 69 | 152 | 346 | 219 | 539 | 60 | 0 | 0 | .. | 188 |
| 2014 | 20403911 | 3880 | 247 | 296 | 975 | 948 | 424 | 82 | 145 | 310 | 225 | 580 | 46 | 0 | 0 | .. | 224 |
| 2015 | 20412978 | 4232 | 263 | 337 | 1079 | 1037 | 438 | 81 | 153 | 370 | 247 | 682 | 50 | 0 | 0 | .. | 275 |
| 2016 | 20427654 | 4348 | 330 | 407 | 1169 | 1131 | 422 | 82 | 129 | 401 | 244 | 687 | 40 | 0 | 0 | .. | 312 |
| 2017 | 20504551 | 4284 | 308 | 383 | 1093 | 1051 | 409 | 76 | 164 | 396 | 225 | 722 | 65 | 0 | 0 | .. | 304 |
| 2018 | 20543000 | 4325 | 323 | 414 | 1055 | 1032 | 439 | 50 | 147 | 395 | 253 | 744 | 55 | 0 | 0 | .. | 280 |
| 2019 | 20488970 | 4165 | 313 | 422 | 972 | 940 | 420 | 82 | 159 | 369 | 238 | 714 | 75 | 0 | 0 | .. | 302 |
| 2020 | 20425787 | 4623 | 412 | 501 | 1059 | 1021 | 411 | 120 | 154 | 561 | 236 | 731 | 56 | 0 | 0 | 73 | 514 |
| 2021 | 21001251 | 5153 | 463 | 612 | 1183 | 1150 | 399 | 90 | 166 | 654 | 289 | 804 | 25 | 0 | 0 | 174 | 590 |
| 2022 | 20741735 | 5073 | 482 | 584 | 1082 | 1050 | 480 | 100 | 171 | 714 | 259 | 711 | 51 | 0 | 0 | 102 | 671 |

**Supplementary Table 12. Negative Binomial Regression Model to assess the association of municipal poverty rate in children and adolescents, and 15-17 male school enrolment rate with homicide rate in the pediatric population in Mexico in 2022.**

|  | **β Coefficient** | **P value** | **Incidence Rate Ratio**  **(95% CI) *** |
| --- | --- | --- | --- |
| **School enrolment rate in 15-17male adolescents (decile) ^¥^** | -0.217 | <0.001 | 0.80  (0.74-0.87) |
| **Decile of children and adolescents poverty rate (decile) ^¥^** | -0.271 | <0.001 | 0.76 (0.70-0.83) |
| **Interaction: school enrolment rate x poverty rates** | 0.016 | 0.04 | 1.02  (1.0-1.03) |
| **Constant** | 3.6 | <0.001 | - |
| School enrolment and poverty rate reported by the National Council for Evaluation of Politics and Development (CONEVAL) in 2020 (11). The model was developed with data from the 1849 municipalities with more than >1600 inhabitants under 20 years old.  *Robust standard error method | | | |
